# Supplementary material for: A “Special” Solvent to Prepare Alloyed Pd2Ni1 Nanoclusters on a MWCNT Catalyst for Enhanced Electrocatalytic Oxidation of Formic Acid
Source: Nanomaterials (Basel). 2023 Feb 17;13(4):755. doi: 10.3390/nano13040755 (PMC9963789; doi:10.3390/nano13040755)
Supplement: Supplementary file 1 [file nanomaterials-13-00755-s001.zip › nanomaterials-2117188-supplementary.pdf]

Supporting Information

A "Special" Solvent to Prepare Alloyed Pd_2Ni_1 Nanoclusters on a MWCNT Catalyst for Enhanced Electrocatalytic Oxidation of Formic Acid

Pingping Yang [1,2,†], Li Zhang [1,2,†], Xuejiao Wei [1], Shiming Dong [1], Wenting Cao [1], Dong Ma [1], Yuejun Ouyang [1],
Yixi Xie [2,*], and Junjie Fei [2,*]

1 College of Chemistry and Materials Engineering, Huaihua University; Huaihua, 418008, Hunan, China yangpingping0218@163.com (P.Y.); zhanglicsu2013@163.com (L.Z.); wei1348137@163.com (X.W.); dsm20032022@163.com (S.D.); cwt1822707678@163.com (W.C.); md12341110@163.com (D.M.); oyyj0816@163.com (Y.O.)
2 College of Chemistry, Xiangtan University, Xiangtan 411105, Hunan, China
* Correspondence: xieyixige@xtu.edu.cn (Y.X.); fei_junjie@xtu.edu.cn (J.F.)
† These authors contributed equally to the work.

Table of Contents:

1. Calculate details

The calculation details are as follows:

"To calculate the average particle size of the alloy, we used ImageJ software to calculate. Firstly, select a TEM image with enough sample size of 100 nm scale bar, enlarge the TEM image to a size that is conducive to measuring the particle size, draw a straight line on the scale bar using the line measurement tool, and calibrate the line to the length of 100 nm of the scale bar; then use the linear measurement tool to randomly measure the particle size of 200 samples in the image; finally, calculate the average particle size of 200 samples. Here we have also added part of the original text: To measure the particle size of the alloy, 200 random samples were chosen from the TEM images." (Paragraph 1 of support information part)

The average size of the alloy particles was calculated to be 3.6 nm (Pd_2Ni_1/CNTs) and 4.1 nm (Pd/CNTs).

2. Supplementary Figures

Figure S1. The corresponding particle size distribution of Pd/CNTs.

Figure S2. TEM and HRTEM images of Pd/CNTs.

Figure S3. EDX line-profiles (a), spot scanning (b) of Pd_2Ni_1 nanoparticle (where Pd is in red and Ni in blue) of Pd_2Ni_1/CNTs.

Figure S4. TEM images and size distribution histograms of Pd_2Ni_1/CNTs (A, B) and Pd/CNTs (C, D) after the current-time measurements in 1.0 M HCOOH + 0.5 M H_2SO_4 solution.

3. Supplementary Tables

Table S1. Elemental composition of the samples obtained from ICP.

Table S2 A recent literatures survey of the activity of FAOR electrocatalysts.

Table S3. Pd 3d peaks of Pd_2Ni_1/CNTs and Pd/CNTs.

4. Supplementary References

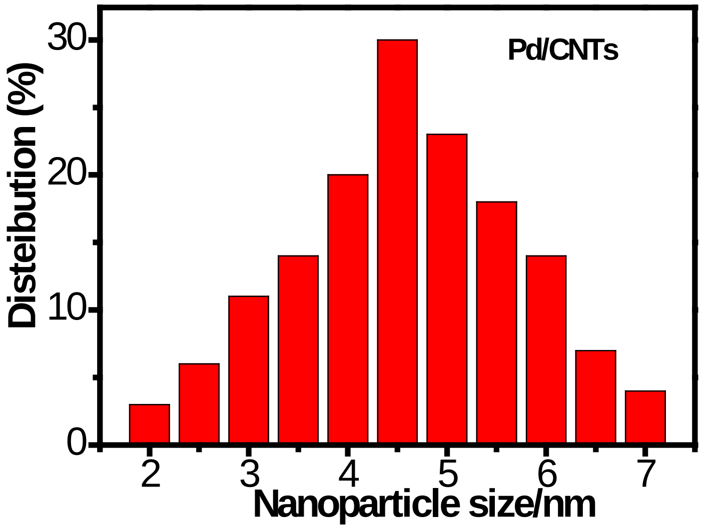

Figure S1. The corresponding particle size distribution of Pd/CNTs.

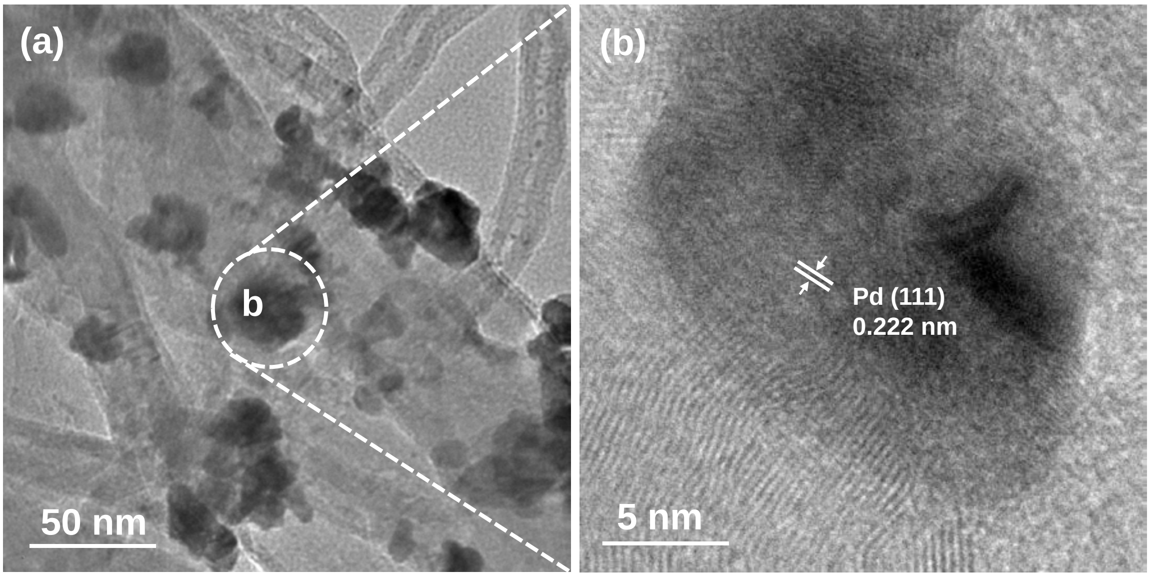

Figure S2. TEM (a) and HRTEM (b) images of Pd/CNTs catalyst.

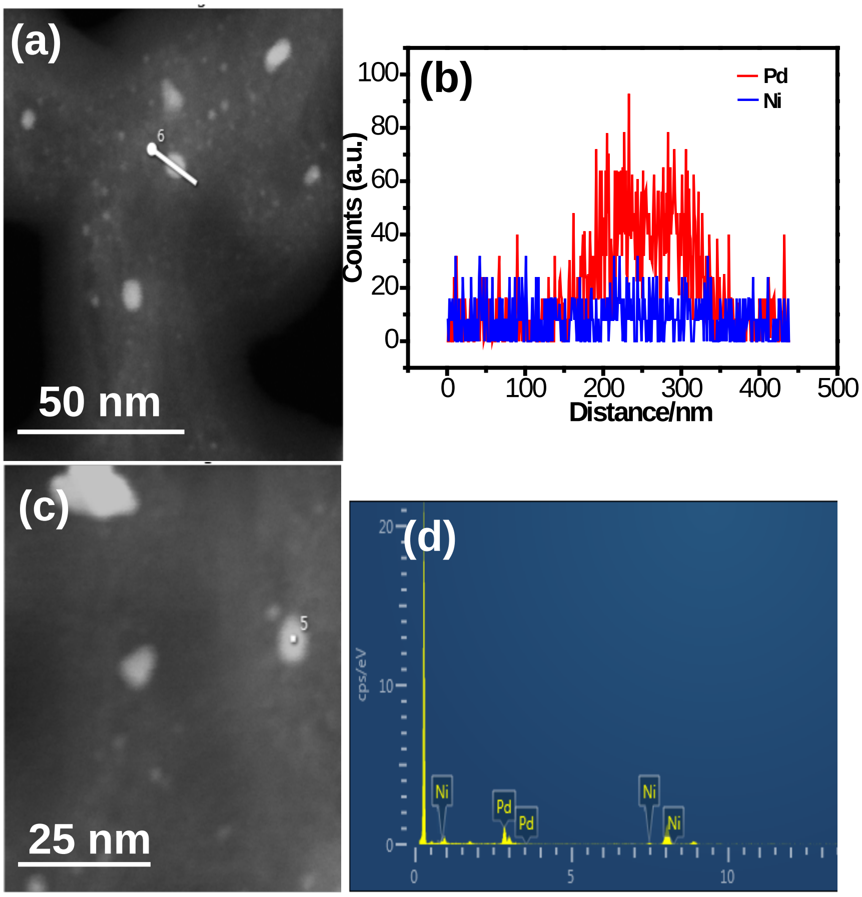

Figure S3. EDX line-profiles (a, b), spot scanning (c, d) of a Pd_2Ni_1 nanocluster (where Pd is in red and Ni in blue).

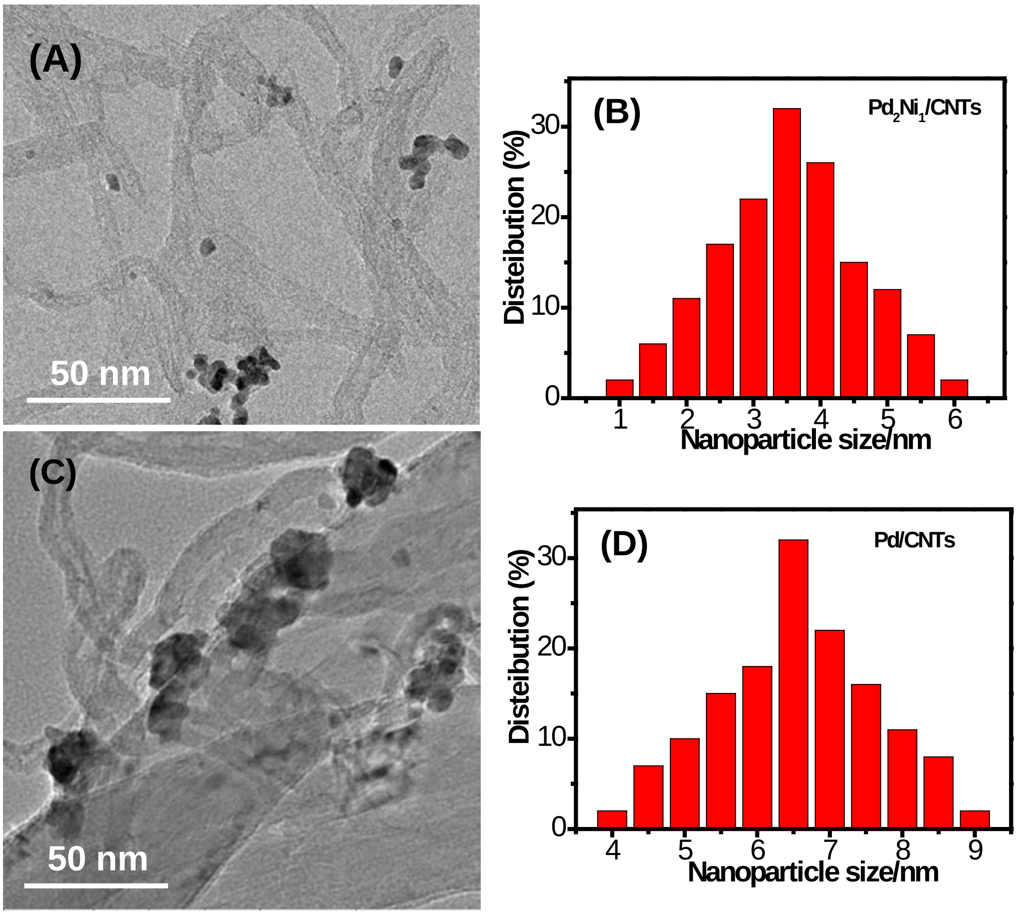

Figure S4 TEM images and size distribution histograms of Pd_2Ni_1/CNTs (A, B) and Pd/CNTs (C, D) after the current-time measurements in 1.0 M HCOOH + 0.5 M H_2SO_4 solution.

Table S1. Pd 3d peaks of Pd_2Ni_1/CNTs and Pd/CNTs.

**Catalysts**	**Pd^0 $3d_{5/2}$ (eV)**	**Pd^0 $3d_{3/2}$ (eV)**	**Pd^{2+} $3d_{5/2}$ (eV)**	**Pd^{2+} $3d_{3/2}$ (eV)**
Pd_2Ni_1/CNTs	335.6	340.8	336.0	341.5
Pd/CNTs	335.8	336.6	341.2	341.6

Table S2 a recent literatures survey of the activity of FAOR electrocatalysts.

**Catalysts**	**Mass activity ($mA\ mg^{-1}{}_{Pd}$)**	**References**
**Pd_2Ni_1/CNTs**	**3351.6**	**This work**
PdCo nanodots	1362.1	[1]
PdCu nanochains	1108.2	[2]
PdCu clusters	1289.0	[3]
coral-like PdCu	1050.0	[4]
Pd_6Co nanocrystals	430.8	[5]
PdCu nanoparticles	194.5	[6]
(3D) porous PdSn	553.4	[7]
PdCu porous network	517.0	[8]
PdeFe nanoparticles	1000.0	[9]
PdCu/CNTs	252.0	[10]

Table S3. Elemental composition of the samples obtained from ICP.

**Elements / Catalysts**	**Pd(wt.%)**	**Ni(wt.%)**	**Atomic ratios**
Pd_2Ni_1/CNTs	18.6	9.31	2:1
Pd_1Ni_1/CNTs	19.2	17.8	1:1
Pd_1Ni_2/CNTs	19.6	38.1	1:2
Pd/CNTs	16.3	-	-

References

[1] Zhang. L-Y, et al. Palladium-cobalt nanodots anchored on graphene: In-situ synthesis, and application as an anode catalyst for direct formic acid fuel cells. Appl Surf Sci 2019, 469, 305-311.

[2] Zhang. L-Y, et al. Twisted palladium-copper nanochains toward efficient electrocatalytic oxidation of formic acid. J Colloid Interf Sci 2019, 537, 366-374.

[3] Zhang. Z, et al. Facile fabrication of stable PdCu clusters uniformly decorated on graphene as an efficient electrocatalyst for formic acid oxidation. Int J Hydrog Energ 2019, 44, 2731-2740.

[4] Zheng J, et al. Coral-like PdCu Alloy Nanoparticles Act as Stable Electrocatalysts for Highly Efficient Formic Acid Oxidation. ACS Sustain Chem Eng 2019, 7, 15354-15360.

[5] Zhang. L-Y, et al. Facile one-pot surfactant-free synthesis of uniform Pd_6Co nanocrystals on 3D graphene as an efficient electrocatalyst toward formic acid oxidation. Nanoscale 2016, 8, 1905-1909.

[6] Wang. L, et al. Pd-Cu/C electrocatalysts synthesized by one-pot polyol reduction toward formic acid oxidation: Structural characterization and electrocatalytic performance. Inter J Hydrog Energ. 2015, 40, 1726-34.

[7] Sun. D-D, et al. Nanobranched porous palladium-tin intermetallics: One-step synthesis and their superior electrocatalysis towards formic acid oxidation. J Power Sources 2015, 280, 141-146.

[8] Yang. F, et al. Pd-Cu alloy with hierarchical network structure as enhanced electrocatalysts for formic acid oxidation. Inter J Hydrog Energ 2016, 41, 6773-6780.

[9] Feng. A-N, et al. Surfactant-free Pd-Fe nanoparticles supported on reduced graphene oxide as nanocatalyst for formic acid oxidation. Inter J Hydrog Energ 2017, 42, 15196-15202.

[10] Zhu. F-C, et al. High activity of carbon nanotubes supported binary and ternary Pd-based catalysts for methanol, ethanol and formic acid electro-oxidation. J Power Sources 2013, 242, 610-20.
